# Deoxybouvardin-glucoside induces apoptosis in non-small cell lung cancer cells by targeting EGFR/MET and AKT signaling pathway

**DOI:** 10.17179/excli2024-7359

**Published:** 2024-10-21

**Authors:** Na Yeong Lee, Sang Hoon Joo, A-Young Nam, Seung-On Lee, Goo Yoon, Seung-Sik Cho, Yung Hyun Choi, Jin Woo Park, Jung-Hyun Shim

**Affiliations:** 1 Department of Biomedicine, Health & Life Convergence Sciences, BK21 Four, College of Pharmacy, Mokpo National University, Muan 58554, Republic of Korea; 2 College of Pharmacy, Daegu Catholic University, Gyeongsan 38430, Republic of Korea; 3 Department of Pharmacy, College of Pharmacy, Mokpo National University, Muan 58554, Republic of Korea; 4 Department of Biochemistry, College of Korean Medicine, Dong-Eui University, Busan 47227, Republic of Korea; 5 The China-US (Henan) Hormel Cancer Institute, Zhengzhou, Henan, 450008, P.R. China

**Keywords:** deoxybouvardin glucoside, non-small cell lung cancer, EGFR/MET/AKT, cell cycle, reactive oxygen species, apoptosis

## Abstract

Non-small cell lung cancer (NSCLC) is a leading cause of cancer-related deaths worldwide. Its treatment is complicated due to the development of resistance to conventional chemotherapy and targeted therapy. Deoxybouvardin and related cyclic hexapeptides reportedly exhibit antitumor activities, but their mechanisms of action remain unclear. This study aimed to investigate the anticancer mechanisms of deoxybouvardin glucoside (DBG), a glucosidic form of deoxybouvardin from* Rubia* species, in gefitinib (GEF)-sensitive and -resistant NSCLC HCC827 cells. The effects of DBG treatment on cell proliferation were evaluated using a viability assay. The inhibitory effects of DBG treatment on the activities and phosphorylation of the protein kinases epidermal growth factor receptor (EGFR), MET, and AKTs were assessed using *in vitro* kinase assay and western blot, respectively. DBG treatment inhibited the growth of HCC827 cells in a concentration- and time-dependent manner. Results of *in vitro* kinase assay and western blotting showed that DBG treatment significantly inhibited the activities and phosphorylation of the protein kinases EGFR, MET, and AKT. Prediction using molecular docking showed that DBG is located in the ATP-binding pockets of these kinases, supporting the kinase inhibition by DBG treatment. Moreover, DBG treatment induced reactive oxygen species (ROS) generation and cell cycle arrest in the cells. The induction of apoptosis by DBG through caspase activation was confirmed by Z-VAD-FMK treatment. In summary, DBG treatment inhibited the growth of GEF-sensitive and -resistant NSCLC cells by targeting EGFR, MET, and AKTs. Moreover, it induced apoptosis by inducing ROS generation and caspase activation. These results indicate that DBG is a potential therapeutic agent for the treatment of GEF-resistant NSCLC.

See also the graphical abstract(Fig. 1).

## Introduction

The global mortality rate of cancer-related diseases remains high with almost two million deaths every year. Lung cancer is the second most common cancer and a leading cause of cancer-related mortality worldwide (Sung et al., 2021[14]). The most common type of lung cancer is non-small cell carcinoma (NSCLC), which is often associated with smoking (Siegel et al., 2022[13]). While the progression of NSCLC is slow, it is often diagnosed only after metastasis to other tissues. Key mutations associated with NSCLC development include mutations in epidermal growth factor receptor (EGFR), rearrangements in anaplastic lymphoma kinase and ROS1, and alterations in MET (Griffin and Ramirez, 2017[4]). Thus, these mutations may serve as chemotherapy targets and diagnostic markers. Gefitinib (GEF) is an EGFR tyrosine kinase inhibitor (TKI) that exerts anticancer activity by inhibiting the EGFR signaling pathway (Kosaka et al., 2011[9]). However, its efficacy in patients with lung cancer is limited by the emergence of acquired resistance (Engelman and Janne, 2008[2]).

Deoxybouvardin glucoside (DBG) is a glycosidic form of deoxybouvardin (DB) initially isolated from the shrub *Bouvardia ternifolia *(Rubiaceae). DB and related cyclic hexapeptides reportedly exhibit antitumor activities (Jolad et al., 1977[5]; Kato et al., 1989[6]). However, their molecular mechanisms of action remain unclear to date.

Thus, this study aimed to explore the antitumor effects of DBG on GEF-sensitive and -resistant NSCLC cell lines HCC827 and HCC827GR, respectively, and elucidate the underlying mechanisms. A viability assay was conducted to evaluate the effects of DBG treatment on the proliferation of the NSCLC cells. *In vitro* kinase assay and molecular modeling were performed to assess the inhibitory effects of DBG treatment on the protein kinases EGFR, MET, and AKTs. Moreover, western blotting was used to analyze the effects of DBG treatment on the phosphorylation of these kinases in the EGFR/MET/AKT pathway. Induction of apoptosis mediated by ROS generation was verified by pretreatment with *N*-acetyl-L-cysteine (NAC). This study suggests DBG as a novel therapeutic agent for the targeted chemotherapy of NSCLC. 

## Materials and Methods

### Chemical

DBG (purity > 95 %) was obtained from Chungnam National University (see Supplementary information, Table 1). 

### Reagents

GEF was obtained from Cayman Chemical Company (Ann Arbor, MI, USA), savolitinib (SAV) and capivasertib (AZD5363) from Selleck Chemicals (Houston, TX, USA), Roswell Park Memorial Institute (RPMI) 1640 medium from Welgene (Gyeongsan, Gyeongbuk, South Korea), Dulbecco's modified Eagle's medium (DMEM) and penicillin/streptomycin (P/S) from Gibco (Carlsbad, CA, US), fetal bovine serum (FBS) from Corning (NY, USA), trypsin 0.25 % (1X) solution from HyClone (Logan, UT, USA), and 3-(4,5-dimethyl-2-thiazolyl)-2,5-diphenyl-2H-tetrazolium bromide (MTT) from VWR International (Radnor, PA, USA), Basal Medium Eagle (BME), sodium bicarbonate, NAC, and Z-Val-Ala-Asp-(OMe)-fluoromethyl ketone (Z-VAD-FMK) were obtained from Sigma-Aldrich (St. Louis, MO, USA). Bacto agar and skim milk were purchased from BD Biosciences (San Jose, CA, US), L-glutamine and gentamicin from Lonza (Walkersville, MD, USA), dimethyl sulfoxide (DMSO) from Duksan (Ansan, Gyeonggi-do, South Korea), and RIPA buffer from iNtRON Biotechnology (Seongnam-si, Gyeonggi-do, South Korea). Primary antibodies against β-actin, cyclin D1, CDK2, CDK4, CDK6, p27, caspase-3, poly(ADP-ribose) polymerase (PARP), Bcl-2, Bad, Bim, cytochrome c (cyto c), β-tubulin, and COX4 were purchased from Santa Cruz Biotechnology (Santa Cruz, CA, USA). Anti-phospho (p)-EGFR (Y1068), anti-EGFR, anti-p-MET (Y1234/1235), anti-MET, anti-p-AKT (S473), and anti-AKT were obtained from Cell Signaling Biotechnology (Beverly, MA, USA). Peroxidase-conjugated secondary antibodies for rabbit and mouse antibodies were obtained from Invitrogen (Carlsbad, CA, USA). Peroxidase-conjugated goat secondary antibodies were purchased from Thermo Fisher Scientific (Waltham, MA, USA). AKT1, AKT2, MET, and EGFR kinase enzyme systems and the ADP-Glo kinase assay were purchased from Promega (Madison, WI, USA).

### Cell culture and treatment

The human keratinocyte cell line HaCaT and human NSCLC cell line HCC827 (EGFR exon 19 deletion, GEF sensitive) were purchased from the American Type Culture Collection (Manassas, VA, USA). HCC827GR (MET amplification, gefitinib resistant) cells were obtained from Professor Pasi A. Jänne and maintained as previously described (Engelman et al., 2007[3]). HaCaT cells were grown in DMEM, whereas HCC827 and HCC827GR cells were grown in RPMI 1640. All culture media were supplemented with FBS (10 %) and P/S (1 %), and the cells were grown at 37 °C in 5 % CO_2_. HCC827GR cells were maintained in medium containing 0.1 µM GEF. HCC827 and HCC827GR cells were treated with different concentrations of DBG (3, 6, and 12 nM) for 48 h. In some experiments, the cells were pretreated with the inhibitor Z-VAD-FMK (12 μM) or NAC (4 mM) for 3 h before treatment with 12 nM DBG for 48 h.

### MTT assay

The MTT viability assay was performed to evaluate the anticancer effects of DBG on NSCLC cells. HCC827, HCC827GR, and HaCaT cells were seeded in 96‐well plates at densities of 6×10^3^, 5.5×10^3^, and 8×10^3^ cells/well, respectively, and then treated with DBG (0, 3, 6, and 12 nM), GEF (1 μM, positive control), or SAV (2 nM, positive control) as indicated for 24 or 48 h. The cells were treated with MTT and incubated for 2 h. The supernatants were removed, and the remaining formazan crystals were dissolved in DMSO before absorbance measurement at 570 nm using a Multiscan GO spectrophotometer (Thermo Fisher Scientific, Vantaa, Finland).

### Soft agar assay

The anchorage-independent cell growth assay was performed in 6-well plates with dual agar layers containing 0.6 % agar (lower layer) and 0.3 % agar (upper layer) supplemented with BME, 2 mM L-glutamine, 5 μg/ml gentamicin, and 10 % FBS. After 2 weeks of incubation, the number of colonies was assessed using a microscope (Leica, Wetzlar, Germany), and the mean colony area was analyzed using i-Solution ^TM^ software (Vancouver, BC, Canada).

### In vitro kinase assay

We performed an *in vitro* kinase assay to evaluate the effects of DBG on EGFR, MET, and AKT. Protein kinase AKT1 (10 ng), AKT2 (5 ng), MET (4 ng), or EGFR (4 ng) was resuspended in each well of a 384-well plate containing DBG (3, 6, and 12 nM), AZD5363 (30 nM) or SAV (2 nM) or GEF (1 μM), substrates (0.2 μg/μl), ATP (150, 250, 10, or 5 μM), and kinase reaction buffer [1 mg/ml BSA, 50 μM DTT, 20 mM MgCl_2_, 2 mM MnCl_2_, 100 μM sodium vanadate, and 40 mM Tris (pH 7.5)] and then incubated at room temperature (RT) for 60 min. Subsequently, each well was added with ADP-Glo reagent and incubated for another 40 min. After completion of the kinase reaction, each well was added with kinase activity detection reagent and then incubated for 30 min. Liquid was dispensed with I.DOT (Dispendix, Stuttgart, Germany). Luminescence was measured on a Centro LB 960 microplate luminometer (Berthold Technologies).

### Molecular modeling

The binding between DBG and protein kinases was predicted by molecular modeling using AutoDock Vina (Trott and Olson, 2010[15]). The structures of EGFR (1M17), MET (4XYF), AKT1 (6CCY), and AKT2 (3D0E) were obtained from Protein Data Bank. The structural data file for DBG was obtained from the PubChem library. The search grid was set to encompass most of the protein surface to ensure an unbiased search. Each AutoDock Vina simulation generated 10 modes. The best mode was used to depict the structural features.

### Western blotting

Western blotting was performed on the NSCLC cells treated with DBG to identify the proteins related to apoptosis and to determine whether the inhibition of kinase activity *in vitro* could be related to the regulation of signaling pathways involving the kinases EGFR, MET, and AKT. Cells were lysed in RIPA buffer, and protein concentration was determined using a Bio-Rad DC Protein Assay kit (Bio-Rad, Hercules, CA, USA). Protein samples from the cell lysates were resolved by sodium dodecyl sulfate-polyacrylamide gel electrophoresis and then transferred onto polyvinylidene fluoride membranes. The membranes were blocked with 3 % or 5 % skim milk and then incubated at 4 °C overnight with the indicated primary antibodies, followed by the corresponding horseradish peroxidase-conjugated secondary antibodies at RT for 2 h. Protein bands were visualized using western blotting luminol reagent solution and ImageQuant LAS 500 (GE Healthcare, Uppsala, Sweden). The density of the bands was quantified using ImageJ software (NIH, Bethesda, MD, USA).

### Cell cycle analysis

Cell cycle distribution analysis was performed using a Muse™ Cell Cycle Kit (MCH100106, Merck Millipore, Billerica, MA) based on propidium iodide (PI) staining. The cells were collected, fixed in 70 % ethanol overnight at -20 °C, and then stained with a Muse™ Cell Cycle Reagent at RT for 30 min in the dark. The cell cycle distribution was measured using a Muse™ Cell Analyzer (Merck Millipore).

### ROS measurement

To elucidate the mechanisms underlying the antiproliferative effects of DBG on NSCLC cells, we analyzed ROS generation in the cells treated with DBG. The levels of intracellular ROS were measured using a Muse™ Oxidative Stress Kit (MCH100111, Merck Millipore) in accordance with the manufacturer's instructions. The cells were incubated with a Muse™ Oxidative Stress Reagent working solution in the dark at 37 °C for 30 min. Intracellular ROS generation was measured using a Muse™ Cell Analyzer.

### Isolation of cytosolic and mitochondrial fractions

The cells were suspended in plasma membrane extraction buffer [250 mM sucrose, 10 mM HEPES (pH 8.0), 10 mM KCl, 1.5 mM MgCl_2_• 6H_2_O, 1 mM EGTA, 1 mM EDTA, 0.1 mM PMSF, 0.01 mg/ml aprotinin, and 0.01 mg/ml leupeptin], and 0.1 % digitonin was added for vortexing (1 min). The supernatant was collected as the cytosolic fraction. The pellet was resuspended in plasma membrane extraction buffer containing 0.5 % Triton X-100. The lysates were centrifuged at 13,000 rpm for 30 min to separate the mitochondrial fraction.

### Annexin V/7-aminoactinomycin D (7-AAD) staining

Apoptosis was measured using a Muse™ Annexin V & Dead Cell Kit (MCH100105, Merck Millipore). The fluorescent signal was analyzed with a Muse™ Cell Analyzer.

### Multi-caspase assay

To further study the antiproliferative activity of DBG in NSCLC cells, we evaluated the activation of caspases using a Muse™ Multi-Caspase Kit (MCH100109, Merck Millipore).

### Statistical analysis

All data are presented as mean ± standard deviation (SD) based on the results of at least three independent experiments. Statistical analyses were conducted using one-way ANOVA, two-way ANOVA, and Student's t-test in GraphPad Prism software (version 9.0; San Diego, CA, USA). Statistical significance compared with the control groups was considered at *p<0.05, **p<0.01, and ***p<0.001.

## Results

### DBG suppresses the proliferation of GEF-sensitive and -resistant NSCLC cells

**Figure 2:**
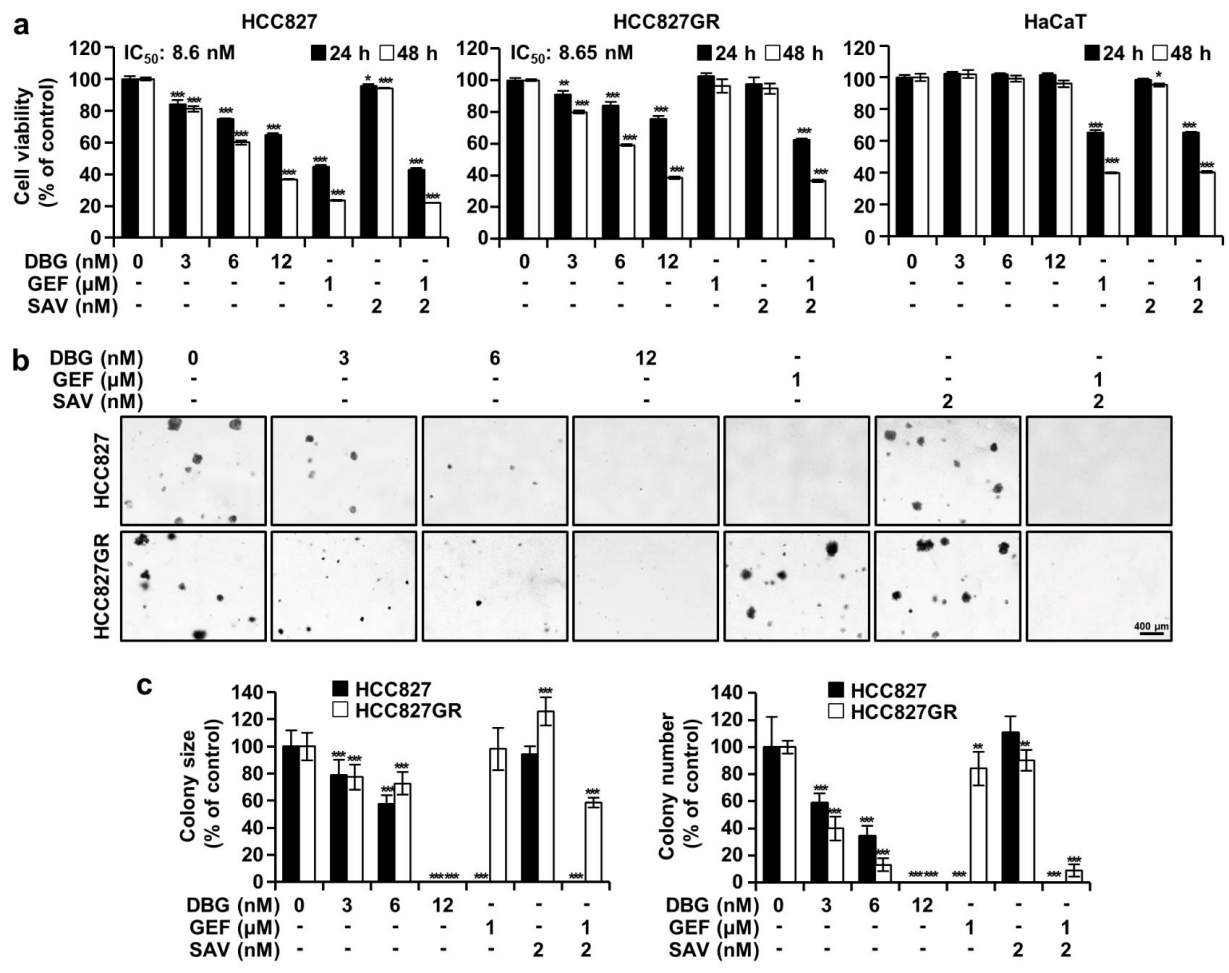
Antiproliferative activity of deoxybouvardin glucoside (DBG) treatment in non-small cell lung cancer (NSCLC) cells. (a) NSCLC (HCC827 and HCC827GR) and HaCaT cells were treated with DBG (0, 3, 6, and 12 nM), gefitinib (GEF, 1 µM), or savolitinib (SAV, 2 nM) as indicated for 24 (black) and 48 h (white). Cell viability determined using the 3-(4,5-dimethyl-2-thiazolyl)-2,5-diphenyl-2H-tetrazolium bromide (MTT) cell viability assay. Data are shown as the mean ± SD (n=3). *p<0.05, **p<0.01, and ***p<0.001 compared with the control group. (b, c) Soft agar assay was performed to determine the anchorage-independent colony growth in the NSCLC cells after the indicated treatments for 2 weeks. (b) HCC827 and HCC827GR cells were observed by microscopy after the indicated treatments for 2 weeks. (c) Size and number of colonies. **p<0.01 and ***p<0.001 compared with the control group.

MTT assay results indicated that treatment with DBG (3, 6, and 12 nM) suppressed the viability of the HCC827 and HCC827GR cells but did not affect that of the HaCaT cells (Figure 2a(Fig. 2)). The IC_50_ values of DBG treatment for 48 h were 8.6 and 8.65 nM for the HCC827 and HCC827GR cells, respectively. After treatment with GEF (1 µM), SAV (2 nM), and their combination for 48 h, the viability rates of the HCC827 cells were 23.44 %, 94.28 %, and 21.95 %, respectively, whereas those of the HCC827GR cells were 96.35 %, 94.70 %, and 36.65 %, respectively. In the HaCaT cells, the corresponding viability rates were 39.87 %, 95.26 %, and 40.29 %. Meanwhile, soft agar assay results showed that after treatment with 3, 6, and 12 nM DBG, the relative colony sizes of the HCC827 cells were 78.77 %, 57.75 %, and 0.00 %, respectively, whereas those of the HCC827GR cells were 77.33 %, 72.67 %, and 0.00 %, respectively (Figure 2b, c(Fig. 2)). After the same treatment, the colony counts of the HCC827 cells were 58.65 %, 34.62 %, and 0.00 % compared with the control, whereas those of the HCC827GR cells were 40.00 %, 13.10 %, and 0.00 %, respectively. After treatment with 1 µM GEF, the HCC827 cells did not form any colonies, whereas the HCC827GR cells showed a colony size and count of 98.13 % and 84.14 %, respectively. Treatment with 2 nM SAV did not significantly alter the colony size and count, with 94.26 % and 110.58 % for the HCC827 cells, respectively, and 125.91 % and 90.34 % for the HCC827GR cells, respectively. After cotreatment with GEF and SAV, the colony size and count of the HCC827GR cells were 58.53 % and 8.97 %, respectively, whereas the HCC827 cells did not form colonies (Figure 2c(Fig. 2)).

### DBG inhibits kinase activity in vitro

**Figure 3:**
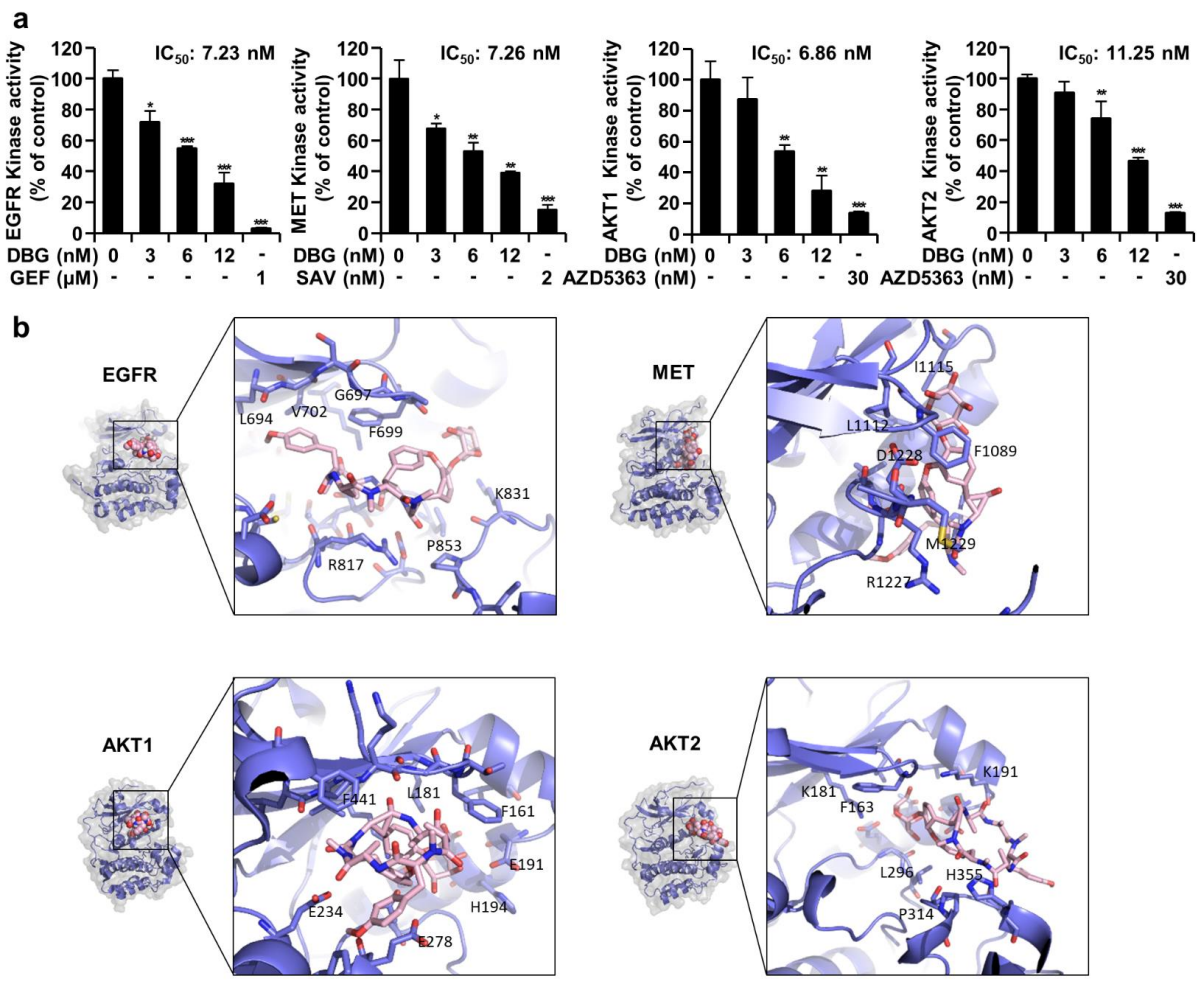
Inhibitory effect of deoxybouvardin glucoside (DBG) treatment on protein kinases. (a) *In vitro *ADP-Glo kinase activity assay for epidermal growth factor receptor (EGFR), MET, AKT1, and AKT2 to determine the inhibitory effects of DBG, gefitinib (GEF), or savolitinib (SAV), or capivasertib (AZD5363). Data are shown as the mean ± SD (n=3). *p<0.05, **p<0.01, and ***p<0.001 compared with the control group. (b) Molecular modeling of DBG binding with protein kinases. Bird's eye view: Protein kinases are shown as surface and cartoon and DBG in spheres. Zoomed in: DBG (pink) and surrounding amino acids (purple)

*In vitro* kinase assay results indicated that treatment with DBG (3, 6, and 12 nM) significantly inhibited the kinase activities of EGFR, MET, AKT1, and AKT2. The degree of inhibition by DBG was similar in these kinases, and the IC_50_ values were 7.23, 7.26, 6.86, and 11.25 nM for EGFR, MET, AKT1, and AKT2, respectively (Figure 3a(Fig. 3)). Supporting the results obtained from the *in vitro* kinase assay, molecular docking results (Trott and Olson, 2010[15]) placed the entire cyclic hexapeptide and glucoside in the ATP-binding pocket of each kinase (Figure 3b(Fig. 3)). Hydrophobic amino acids, including Leu694, Ala698, Phe699, Val702, and Pro853, in EGFR surrounded DBG in this mode, and similar hydrophobic interactions were found in other proteins.

### DBG regulates the signaling pathway of EGFR, MET, and AKT

**Figure 4:**
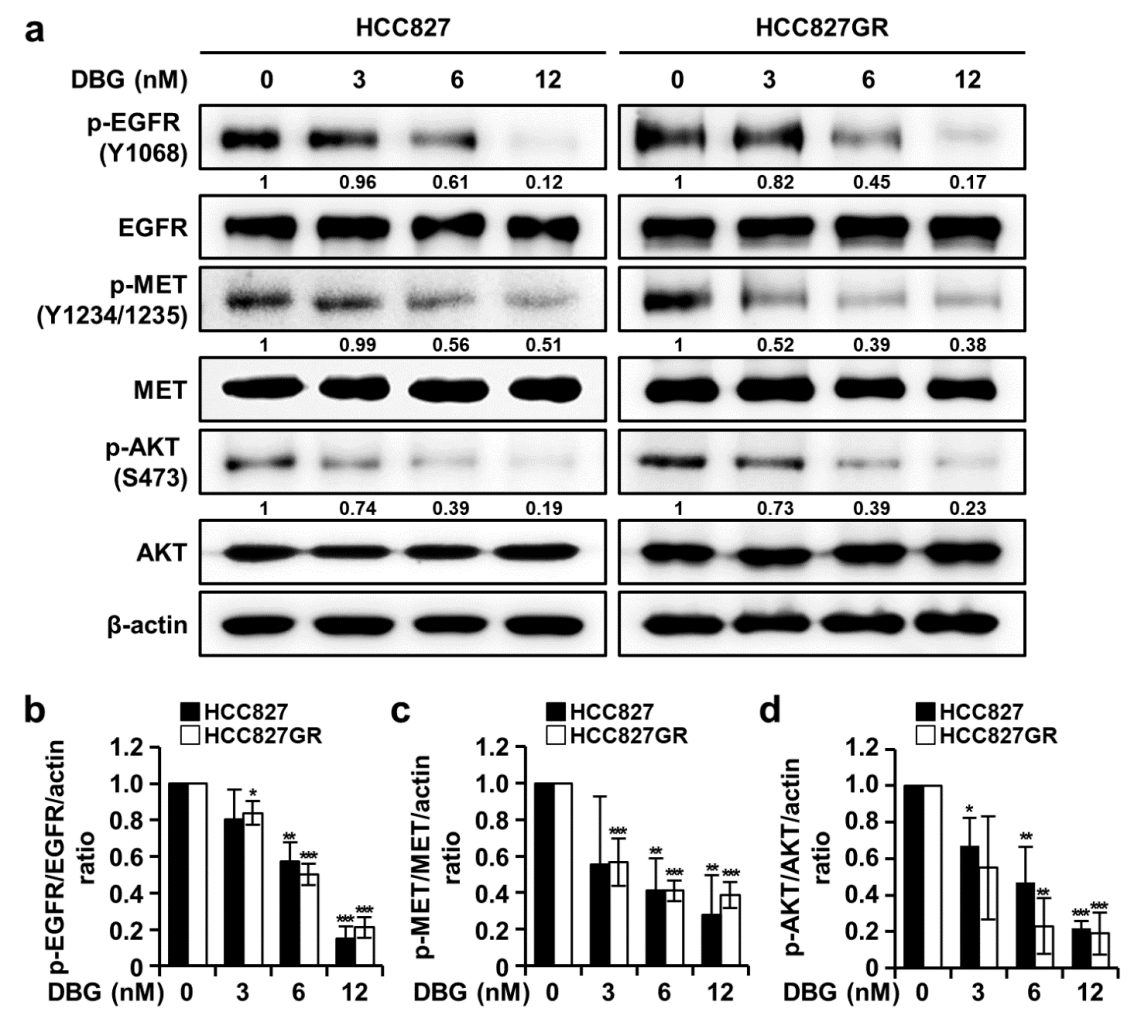
Inhibitory effects of deoxybouvardin glucoside (DBG) treatment on epidermal growth factor receptor (EGFR), MET, and AKT signaling. (a) Non-small cell lung cancer (NSCLC) cells HCC827 and HCC827GR were treated with DBG (0,3, 6, and 12 nM) for 48 h before western blotting to detect p-EGFR (Y1068), EGFR, p-MET (Y1234/1235), MET, p-AKT (S473), and AKT. β-actin was used as the loading control. (b, c, d) Histogram representing the densitometric analysis of protein expression. Data are shown as the mean ± SD (n=3). *p<0.05, **p<0.01, and ***p<0.001 compared with the control group

Western blotting results of the changes in the protein and phosphorylation levels of the kinases EGFR, MET, and AKT are shown in Figure 4(Fig. 4). The phosphorylation levels of the kinases decreased in the HCC827 and HCC827GR cells treated with DBG. These results suggest that DBG regulates the signaling pathways of EGFR, MET, and AKT.

### DBG modulates the cell cycle transition of NSCLC cells at the G_0_/G_1_ phase

**Figure 5:**
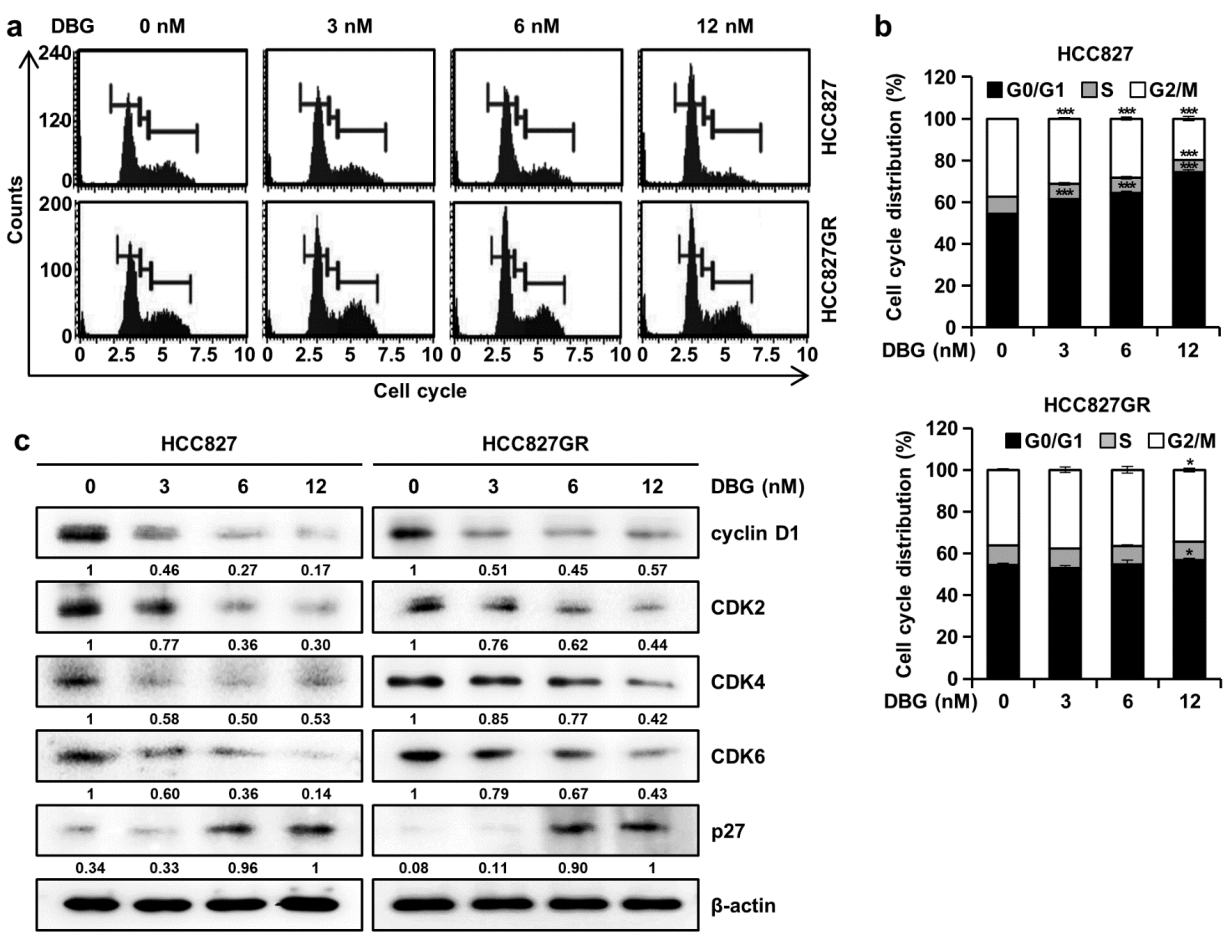
Cell cycle modulation by deoxybouvardin glucoside (DBG). Non-small cell lung cancer (NSCLC) cells HCC827 and HCC827GR were treated with DBG (0, 3, 6, and 12 nM) for 48 h before flow cytometry with PI staining. (a) Flow cytometry plots. (b) Cell cycle distribution. *p<0.05, and ***p<0.001 compared with the control group. (c) Immunoblot of proteins related with cell cycle regulation cyclin D1, CDK2, CDK4, CDK6, and p27. β-actin was used as the loading control.

Treatment with DBG increased the proportions of HCC827 and HCC827GR cells at the G_1_ phase by 19.94 % and 2.36 %, respectively (Figure 5a, b(Fig. 5)). We suspected that DBG treatment caused cell cycle arrest at the G_0_/G_1 _phase. Treatment with DBG also significantly decreased the levels of cyclin D1 and CDKs in a dose-dependent manner (Figure 5c(Fig. 5)). These data indicate that DBG induces cell cycle arrest at the G_1_ phase and exerts antiproliferative effects.

### DBG induces ROS generation

**Figure 6:**
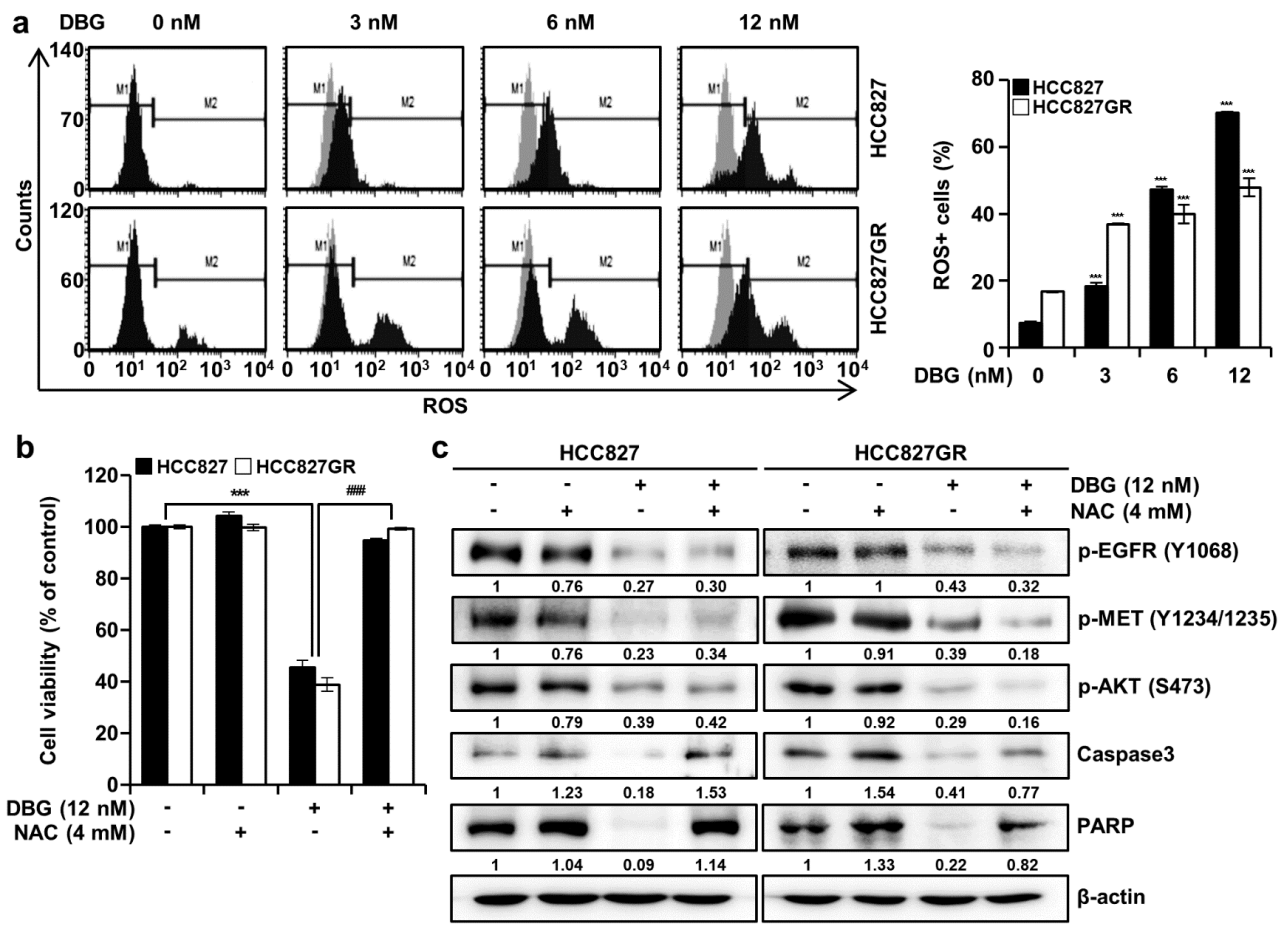
Reactive oxygen species (ROS) generation induction by deoxybouvardin glucoside (DBG) treatment. Non-small cell lung cancer (NSCLC) cells HCC827 and HCC827GR were treated with DBG (0, 3, 6, and 12 nM) for 48 h before flow cytometry with a Muse™ Oxidative Stress Kit. (a) Flow cytometry plots and cellular ROS levels. ***p<0.001 compared with the control group. (b, c) NSCLC cells pretreated with *N*-acetyl-L-cysteine (NAC, 4 mM) or vehicle were incubated with DBG for 48 h. (b) Cell viability was measured using the 3-(4,5-dimethyl-2-thiazolyl)-2,5-diphenyl-2H-tetrazolium bromide (MTT) assay. ***p<0.001 compared with the control group. ^###^*p*<0.001 compared with the DBG treatment. (c) Immunoblot analysis of phosphoproteins p-epidermal growth factor receptor (EGFR), p-MET, p-AKT and proteins caspase-3 and poly(ADP-ribose) polymerase (PARP). β-actin was used as the control.

Treatment with 3, 6, and 12 nM DBG increased the ROS levels in the HCC827 cells from 7.32 % to 18.40 %, 47.32 %, and 70.22 %, respectively, and from 16.69 % to 36.89 %, 39.97 %, and 47.92 %, respectively, in the HCC827GR cells (Figure 6a(Fig. 6)). In addition, we determined the antiproliferative activity of DBG in the NSCLC cells pretreated with NAC, an inhibitor of cellular ROS, by analyzing the viability of cells and the phosphorylation levels of EGFR, MET, and AKT. The anticancer activity of DBG decreased in the HCC827 and HCC827GR cells pretreated with NAC. DBG exhibited a little, if any, cytotoxicity only at 12 nM when the cells were pretreated with 4 mM NAC (Figure 6b(Fig. 6)). However, the decreased phosphorylation of EGFR, MET, and AKT induced by DBG treatment did not change in the cells pretreated with NAC. Furthermore, pretreatment with NAC significantly prevented the increase in cleaved PARP and caspase-3 levels (Figure 6c(Fig. 6)).

### DBG modulates apoptosis related proteins in NSCLC cells

**Figure 7:**
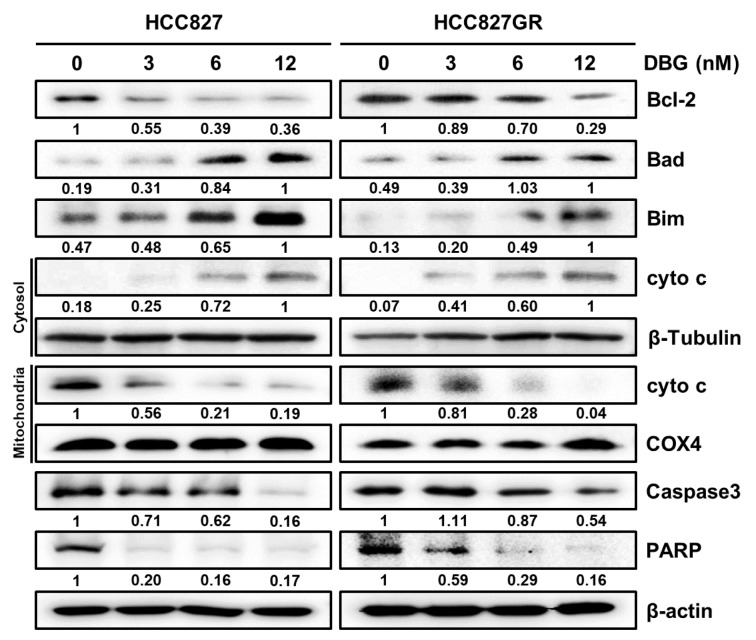
Immunoblot analysis of proteins Bcl-2, Bad, Bim, cytochrome c (cyto c), caspase-3, and poly(ADP-ribose) polymerase (PARP). Cytosol and mitochondrial fractions were separately subjected to immunoblot for cyto c levels. β-Tubulin and COX4 were used as the loading controls for the cytosolic and mitochondrial fractions, respectively.

Western blotting analysis of cellular proteins was performed in NSCLC cells treated with DBG to determine the proteins related to apoptosis. It was observed that the level of antiapoptotic protein Bcl-2 was downregulated in NSCLC cells by DBG treatment, whereas the level of Bad and Bim increased (Figure 7(Fig. 7)). In addition, the analysis of cellular and mitochondrial fractions for cytochrome c indicated that cytochrome c was released from the mitochondria by DBG treatment (Figure 7(Fig. 7)). Moreover, the protein level of whole caspase-3 and PARP decreased in both HCC827 and HCC827GR cells upon DBG treatment.

### DBG induces the apoptosis of NSCLC cells mediated by caspase activation

**Figure 8:**
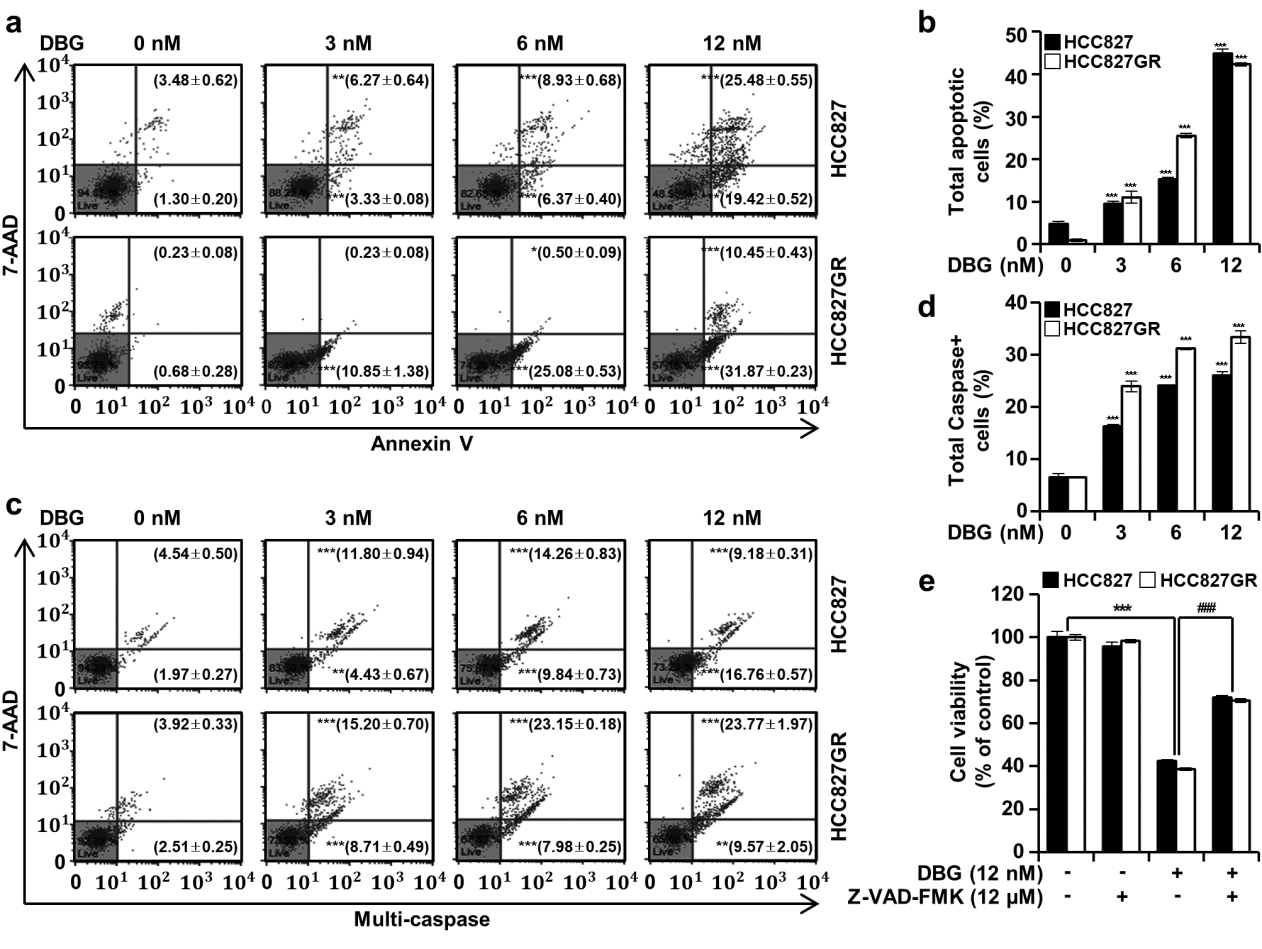
Deoxybouvardin glucoside (DBG)-induced apoptosis mediated by caspase activation. Non-small cell lung cancer (NSCLC) cells HCC827 and HCC827GR were treated with DBG (0, 3, 6, and 12 nM) for 48 h before flow cytometry with annexin V/7-AAD double staining and a Muse™ Multi-Caspase Kit. (a) Annexin V/7-AAD double staining assay. (b) Proportion of apoptotic cells. ***p<0.001 compared with the control group. (c) Multi-caspase analysis. (d) Proportion of cells with caspase activation. ***p<0.001 compared with the control group. (e) NSCLC cells pre- or cotreated with Z-VAD-FMK (12 µM) or vehicle for 3 h were incubated with DBG for 48 h. Cell viability was measured using the 3-(4,5-dimethyl-2-thiazolyl)-2,5-diphenyl-2H-tetrazolium bromide (MTT) assay. ***p<0.001 compared with the control group. ^###^*p*<0.001 compared with the DBG treatment group

To further study the antiproliferative activity of DBG in NSCLC cells, HCC827 and HCC827GR cells treated with DBG at 3, 6, and 12 nM were analyzed by flow cytometry with annexin V/7-AAD double staining and Muse™ Multi-Caspase reagent staining. The population of apoptotic cells, annexin V + in the flow cytometry plot, increased from the background 4.78 % to 9.60, 15.30, and 44.90 % in HCC827 cells (Figure 8a, b(Fig. 8)). In HCC827GR cells, the apoptotic population increased from 0.92 % to 11.08, 25.58, and 42.32 % (Figure 8a, b(Fig. 8)). The proportion of the cells with activated caspases increased from the background 6.52 % to 16.23, 24.10, and 25.94 % in HCC827 cells (Figure 8c, d(Fig. 8)). The caspase + proportion of HCC827GR cells increased from 6.43 % to 23.91, 31.13, and 33.34 % by DBG treatment (Figure 8c, d(Fig. 8)). The DBG-mediated inhibition in cell viability was significantly recovered by the pan caspase inhibitor Z-VAD-FMK (Figure 8e(Fig. 8)).

See also the supplementary data.

## Discussion

DB and other related bicyclic hexapeptides have shown antitumor activities in cell culture and animal models (Jolad et al., 1977[5]; Zalacain et al., 1982[20]). The rigid structure of cyclic peptides confers structural constraints that allow tight binding to other molecules (Choi and Joo, 2020[1]). An early study suggested that the antitumor activity of DB is mediated by the inhibition of protein synthesis through ribosome binding, but the DB concentration used was in the micromolar range (Zalacain et al., 1982[20]). In the present study, DBG exerted its antitumor activity in the nanomolar range. This study is the first to report the antitumor activity of DBG in GEF-sensitive and -resistant NSCLC cells.

NSCLC treated with an EGFR GEF inhibitor is prone to relapse because of resistance development (Kobayashi et al., 2005[8]). Surprisingly, drug resistance may exist even before chemotherapy is implemented (Wang et al., 2019[16]), impeding therapeutic efficiency. The threonine-to-methionine substitution at amino acid position 790 of EGFR is a principal cause of resistance to EGFR TKI (Kobayashi et al., 2005[8]). The mutated EGFR binds tightly to ATP, thereby weakening the binding affinity of EGFR TKI (Saldana-Rivera et al., 2019[12]). Another frequent alteration in EGFR is *MET *amplification. In the absence of EGFR signaling, survival signaling through the MET kinase may function independently to keep cancer cells alive and proliferating (Peng et al., 2021[11]). Several studies have pursued the simultaneous dual targeting of EGFR and MET to overcome resistance in NSCLC treatment (Lee et al., 2023[10]; Yang et al., 2021[18]; Zhao et al., 2024[21]).

In the present study, DBG treatment inhibited the proliferation of GEF-sensitive and -resistant HCC827 cells (Figure 2(Fig. 2)). The antiproliferative activity of DBG (12 nM) was similar to that of GEF (1 μM) alone in the HCC827 cells and that of GEF and SAV coadministration in the HCC827GR cells, implying that DBG could serve as a chemotherapeutic agent in treating NSCLC.

The survival signaling pathways of EGFR and MET are involved in AKT activation (Kim et al., 2016[7]). Therefore, AKT is a possible target for anticancer therapy. In the present study, DBG treatment inhibited the activities of EGFR, MET, and AKTs in the *in vitro* kinase assay (Figure 3a(Fig. 3)), and the molecular docking model predicted DBG in the ATP-binding pockets of these kinases (Figure 3b(Fig. 3)). It also decreased the phosphorylation levels of these protein kinases in the NSCLC cells (Figure 4a, b(Fig. 4)). However, whether DBG is a direct inhibitor of these protein kinases remains to be clarified.

The cytotoxicity of anticancer therapeutics may involve cell cycle regulation. The anticancer activity of GEF is related to cell cycle arrest at G_0_/G_1_ transition (Wu et al., 2014[17]). In the present study, DBG treatment induced the accumulation of cells at the G_0_/G_1_ phase (Figure 5a, b(Fig. 5)). These results indicate that DBG inhibits cell cycle progression to the G_1_/S phase by decreasing cyclin D1 and CDKs and increasing p27.

DBG treatment increased the levels of cellular ROS in the NSCLC cells (Figure 6a(Fig. 6)). Considering that pretreatment with NAC (4 mM) inhibited ROS generation (Figure 6b(Fig. 6)), we suspected that ROS generation is an upstream signaling event in the DBG-induced apoptosis. Notably, NAC pretreatment did not reverse the decreased phosphorylation levels of EGFR, MET, and AKTs. This result implied that the downregulation of these kinases was an upstream event of ROS generation in the DBG-induced apoptosis (Figure 6c(Fig. 6)). Excessive ROS generation results in the depolarization of MMP (Zorov et al., 2014[22]) and apoptosis (Yang et al., 2020[19]). In the present study, DBG treatment resulted in the release of cyto c, followed by a decrease in the protein levels of PARP and caspase-3 (Figure 7(Fig. 7)), increase in the proportion of apoptotic cells (Figure 8a, b(Fig. 8)), and activation of multiple caspases (Figure 8c, d(Fig. 8)). Pretreatment with Z-VAD-FMK prevented the DBG-induced apoptosis (Figure 8e(Fig. 8)), indicating that the DBG-induced apoptosis was mediated by caspase activation.

Taken together, the results of this study demonstrated that DBG treatment inhibited the growth of GEF-sensitive and -resistant NSCLC HCC827 cells. The *in vitro* kinase assay showed that DBG treatment inhibited the activities of EGFR, MET, and AKTs and suppressed the signaling pathways mediated by these protein kinases. Moreover, DBG treatment induced cell cycle arrest, ROS generation, and caspase activation. Further studies are warranted to identify the molecular targets of DBG and overcome chemotherapy resistance in lung cancer. 

## Notes

Na Yeong Lee and Sang Hoon Joo contributed equally as first author.

Jin Woo Park and Jung-Hyun Shim (Department of Biomedicine, Health & Life Convergence Sciences, BK21 Four, College of Pharmacy, Mokpo National University, Muan 58554, Republic of Korea, Tel: +82-61-450-2684; E-mail: s1004jh@gmail.com) contributed equally as corresponding author.

## Declaration

### Conflicts of interest

The authors claim no conflicts of interest.

### Funding

This work was supported by the National Research Foundation of Korea (NRF) grants funded by the Korea government (MSIT) (2022R1A5A8033794, and RS-2024-00336900).

### Author contribution

Na Yeong Lee: Investigation, writing - original draft. Sang Hoon Joo: Conceptualization, writing - review & editing. A-Young Nam: Investigation. Seung-On Lee: Investigation. Goo Yoon: Validation, resources. Seung-Sik Cho: Validation, resources. Yung Hyun Choi: Validation, resources. Jin Woo Park: Supervision, funding acquisition. Jung-Hyun Shim: Project administration, supervision, funding acquisition, writing - review & editing.

### Supporting information

Deoxybouvardin glucoside: yellowish amorphous powder; ESIMS, *m/z* 919.3 [M + H]^+^,* m/z* 941.3 [M + Na]^+^, *m/z* 917.3 [M - H]^-^; ^1^H and ^13^C NMR data (see Supplementary information, Table 1).

## Supplementary Material

Supplementary information

Supplementary data

## Figures and Tables

**Figure 1:**
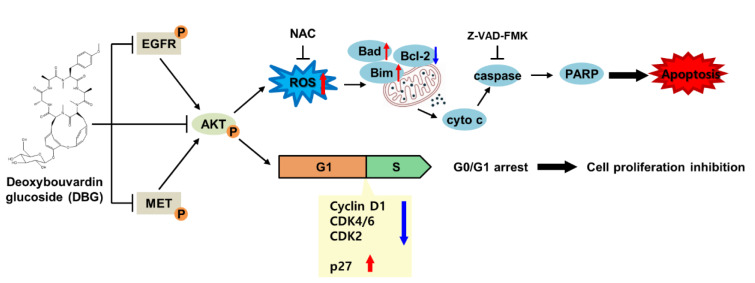
Graphical abstract: The apoptotic mechanism of deoxybouvardin glucoside (DBG) in non-small cell lung cancer cells as an inhibitor of kinases EGFR, AKT, and MET. The inhibition of these kinases leads to the generation of reactive oxygen species, permeabilization of mitochondrial outer membrane, cytochrome c release, caspase activation to induce apoptosis. In addition, cell cycle progress is arrested at the G_0_/G_1_ phase resulting in the inhibition of cell proliferation.
