# Supplementary material for: Deoxybouvardin-glucoside induces apoptosis in non-small cell lung cancer cells by targeting EGFR/MET and AKT signaling pathway
Source: EXCLI J. 2024 Oct 21;23:1287–302. doi: 10.17179/excli2024-7359 (PMC11579512; doi:10.17179/excli2024-7359)
Supplement: Supplementary information [file EXCLI-23-1287-s-001.pdf]

EXCLI Journal 2024;23:1287-1302 – ISSN 1611-2156
Received: May 12, 2024, accepted: October 04, 2024, published: October 21, 2024

Original article:

DEOXYBOUVARDIN-GLUCOSIDE INDUCES APOPTOSIS IN NON-SMALL CELL LUNG CANCER CELLS BY TARGETING EGFR/MET AND AKT SIGNALING PATHWAY

Na Yeong Lee[1,#], Sang Hoon Joo[2,#], A-Young Nam[1], Seung-On Lee[1], Goo Yoon[3], Seung-Sik Cho[1,3], Yung Hyun Choi[4], Jin Woo Park[1,3,*], Jung-Hyun Shim[1,3,5,*]

[1] Department of Biomedicine, Health & Life Convergence Sciences, BK21 Four, College of Pharmacy, Mokpo National University, Muan 58554, Republic of Korea
[2] College of Pharmacy, Daegu Catholic University, Gyeongsan 38430, Republic of Korea
[3] Department of Pharmacy, College of Pharmacy, Mokpo National University, Muan 58554, Republic of Korea
[4] Department of Biochemistry, College of Korean Medicine, Dong-Eui University, Busan 47227, Republic of Korea
[5] The China-US (Henan) Hormel Cancer Institute, Zhengzhou, Henan, 450008, P.R. China

[#] These authors contributed equally to this work as co-first authors.

[*] **Corresponding authors:** Jung-Hyun Shim, Department of Biomedicine, Health & Life Convergence Sciences, BK21 Four, College of Pharmacy, Mokpo National University, Muan 58554, Republic of Korea, Tel: +82-61-450-2684, E-mail: s1004jh@gmail.com
Jin Woo Park, Department of Biomedicine, Health & Life Convergence Sciences, BK21 Four, College of Pharmacy, Mokpo National University, Muan 58554, Republic of Korea, Tel: +82-61-450-2704, E-mail: jwpark@mokpo.ac.kr

https://dx.doi.org/10.17179/excli2024-7359

This is an Open Access article distributed under the terms of the Creative Commons Attribution License (http://creativecommons.org/licenses/by/4.0/).

EXCLI Journal 2024;23:1287-1302 – ISSN 1611-2156
Received: May 12, 2024, accepted: October 04, 2024, published: October 21, 2024

Supplementary Table 1: ^{1}H NMR and ^{13}C NMR spectroscopy data of deoxybouvardin glucoside (in CD_3OD, 300 and 75 MHz)

Position		*^{1}H*	*^{13}C*
Ala1	α	4.48 (m)	48.1
	β	1.26 (ov)	21.0
	C=O		173.4
Ala2	α	4.76 (ov)	45.6
	β	1.33 (ov)	16.4
	C=O		174.6
Tyr3	α	3.73 (ov)	68.3
	β	3.33 (m)	33.7
	γ		132.3
	δ*2	7.10 (ov)	131.9
	ε*2	6.68 (d, *J* = 8.1 Hz)	115.0
	ζ		160.0
	C=O		171.0
	NMe	2.94 (s)	40.3
	OMe	3.80 (s)	55.7
Ala4	α	4.76 (ov)	47.7
	β	1.12 (d, *J* = 6.6 Hz)	18.8
	C=O		173.0
Tyr5	α	5.48 (dd, *J* = 11.4, 9.3 Hz)	55.7
	βa	2.70 (d, *J* = 11.4 Hz)	37.4
	βb	3.60 (ov)	
	γ		137.0
	δa	7.18 (ov)	134.1
	δb	7.53 (ov)	131.9
	εa	7.33 (ov)	127.3
	εb	6.83 (m)	125.1
	ζ		159.8
	C=O		171.3
	NMe	3.09 (s)	31.1
Tyr6	α	4.65 (ov)	58.7
	βa	3.17 (ov)	36.6
	βb	3.48 (ov)	
	γ		131.5
	δa	6.84 (dd, *J* = 8.6, 2.6 Hz)	122.6
	δb	3.80 (s)	115.8
	εa	7.10 (ov)	118.7
	εb		154.7
	ζ		145.4
	C=O		172.2
	NMe	2.65 (s)	30.1
Glucose	1'	5.02 (d, *J* = 7.7 Hz)	102.9
	2'	3.57 (m)	74.9
	3'	3.51 (m)	77.9
	4'	3.45 (m)	71.4
	5'	3.79 (ov)	78.2
	6'	3.89 (ov)	62.5
